# Perspectives on *Yoga* Inputs in the Management of Chronic Pain

**DOI:** 10.4103/0973-1075.63127

**Published:** 2010

**Authors:** Nandini Vallath

**Affiliations:** Consultant, Bangalore Institute of Oncology, Karunashraya Hospice, Bangalore, Karnataka, India

**Keywords:** *Asana*, Experience, Pain, Pranayama, *Yoga*

## Abstract

Chronic pain is multi-dimensional. At the physical level itself, beyond the nociceptive pathway, there is hyper arousal state of the components of the nervous system, which negatively influences tension component of the muscles, patterns of breathing, energy levels and mindset, all of which exacerbate the distress and affect the quality of life of the individual and family. Beginning with the physical body, *Yoga* eventually influences all aspects of the person: vital, mental, emotional, intellectual and spiritual. It offers various levels and approaches to relax, energize, remodel and strengthen body and psyche. *The asanas* and *pranayama* harmonize the physiological system and initiate a “relaxation response” in the neuro endocrinal system. This consists of decreased metabolism, quieter breathing, stable blood pressure, reduced muscle tension, lower heart rate and slow brain wave pattern. As the neural discharge pattern gets modulated, hyper arousal of the nervous system and the static load on postural muscle come down. The function of viscera improves with the sense of relaxation and sleep gets deeper and sustained; fatigue diminishes. Several subtle level notional corrections can happen in case the subject meditates and that changes the context of the disease, pain and the meaning of life. Meditation and *pranayama*, along with relaxing *asanas*, can help individuals deal with the emotional aspects of chronic pain, reduce anxiety and depression effectively and improve the quality of life perceived.

## INTRODUCTION

Acute pain is protective. Chronic pain is no longer a symptom. It lasts beyond natural healing periods, is out of proportion to the causative factor, accompanied by a sensitized nervous system disallowing natural checks; and serves no useful purpose. There are several chemical, anatomical, patho-physiological and genetic alterations in the neural pathways that lead to persisting patterns of pain.[1] It takes characteristics of a disease that needs specific management.

The International Association for Study of Pain (IASP) defines pain as: A subjective unpleasant sensory, emotional experience due to an actual or potential injury or described in terms of such injury). It acknowledges the mind-body aspects of the experience by qualifying it as subjective, unpleasant, sensory and emotional. The experience occurs first in our consciousness and then reflects on to the physiological and behavioral planes. Health professionals can effectively use this knowledge of relationship in understanding and caring for patients with chronic pain.

## BACKGROUND ON *YOGA*

*Yoga* is defined in the *Pathanjali Yoga Suthra* 1: 2 as “annulling the ripples of the mind” 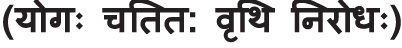


In *Bhagavat Gita* VI 23, it is described as “the discipline which severs the connection with that which causes suffering” 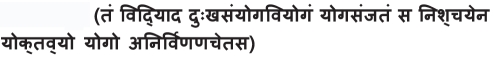


*Yoga* offers various levels of approaches to relax, energize, remodel and strengthen the body and psyche. Beginning with the physical body, which for most people is a practical and familiar starting point, it eventually influences all aspects of the person: vital, mental, emotional intellectual and spiritual. Though there are different personality based paths described traditionally, [*Karma yoga, Jnana yoga, Bhakti yoga* etc] we shall focus our discussion on the most commonly taught method in modern times, R*aja Yoga*.

One of the most systematic collation and compilations of R*aja Yoga* practices was done thousands of years ago by Rishi Pathanjali in his classic work *“Yoga sutras”*. This system has eight limbs of practice [*Ashtanga Yoga*]

It begins with awareness and cultivation of ethical disciplines, codes of behavior and self awareness at personal & interpersonal levels.

Personal: *Yama: Sathya*- Truthfulness in thought, speech and action. This is the highest code of morality. *Ahimsa* – is non violence in thought, speech and action. It is essentially based on love. The cultivation of this quality takes away fear and anger from within. *Brahmacharya* – every interaction merged in the universal consciousness and awareness of divinity. *Astheya*- non stealing/ non coveting. Freedom from craving brings peace. *Aparigraha* – non hoarding. The principle is to take only that which is necessary from nature / world and live in simplicity.

Interpersonal: *Niyama: Shouchya*- purity and discipline in thought, speech and action. *Santosha* - contentment. A mind that is content can concentrate. *Tapas* – austerity. Focusing and channelizing energy i.e. a burning effort to achieve a goal. *Swadhyaya*- cultivating self awareness through contemplation. This brings in the realization that the energy which moves within is the same that moves the entire universe. The identification of microcosm with macrocosm unfolds. *Eshwara Pranidhana* – continuous awareness of divine grace.

These initial steps are lesser known. By reducing the conflicts within and bringing about clarity in priorities and purpose of life, these disciplines gradually reduce the frequency of stressful interactions in the practitioner’s life. Commitment to thought, purity improves the spiritual quotient of the person, harmonizes his existence within the society and most importantly maintains the stability of his biological homeostatic systems through a stabilized neuro-endocrine flow.

Then comes the better known aspects, the postures [*asanas*], cleansing practices [*kriyas*], control of vital energy rhythms [*pranayama*] and meditation. The evolution in practice gradually develops according to individual efforts and commitment.

## MULTIDIMENSIONALITY OF CHRONIC PAIN

Chronic pain is often described in terms of total pain, with physical, emotional, social and spiritual components. The discussion below is to reiterate the contribution of physical and non physical aspects for perpetuation of chronic pain.

### Physical

Chronic pain is not just a symptom. It takes the form of disease through active processes, in the form of multiple plastic changes that together determine the duration and intensity of the pain.[2] There is central and peripheral sensitization in the nervous system with neuro-chemical and neuro-anatomical components that eventually results in hyperexcitability, recruitment and disinhibition of the pathways, that amplifies and perpetuates chronic pain.

Besides the actual nociceptive initiators, there are other strong neuro-endocrine-immunologic components to chronic pain perceptions. The breathing becomes mainly thoracic with lesser excursion of the diaphragm.[3] There is sustained deep muscle tension characterized by repetitive static loads to the muscles and psychological stress, especially of the postural groups of muscles.[4]

### Emotional

Chronic pain decreases function and causes anxiety and depression.[5] The physiology is in a state of persistent stress with the hypothalamic – pituitary- adrenal axis and the sympathetic system, in a state of hyper arousal. It affects sleep, energy reserve and appetite adversely.[6] High scores on a psychosocial screening and emotional distress (Hopkin’s symptom check list) during the acute episode were also significantly associated with non-recovery and persistence of pain.[7]

### Social

Interpersonal relations, which are a crucial part of social interactions for all human beings, are often adversely influenced by chronic pain. With constant unremitting pain, the sufferer feels loss of control over the situation looses self confidence and withdraws. Some may become irritable, hostile and aggressive. People who are close to the ill person could react with over protectivity, avoidance, guilt or resentment.

The loss of role, the fear of dependency and future sufferings continuously perturb the chronic pain patient. There is a sense of isolation and loneliness. There is evidence to suggest inadequate social problem solving as an important factor in the pathogenesis of chronic pain.[8,9]

## INTELLECTUAL AND SPIRITUAL

The persistent pain, with its uncertainty of progress, deep distress and interpretations creates a sense of hopelessness, helplessness, lack of meaning for what is happening, guilt or disturbed belief systems.

The five dimensions together evolve into a state of total pain with persistent, disturbing ripples in the mind, which maintains the neural wind up and lowers the threshold of pain perception further.

### How do asanas help in chronic pain conditions?

Asanas, the yogic postures, are popularly identified as ‘The *Yoga*’ by the lay person. It is but, one of the eight limbs of *yoga* philosophy. There are different types of *asanas*: physical culturing, balancing, relaxing and meditative.

### Effect of asanas on the musculo-skeletal system

In most chronic pain conditions, whether they are directly related to musculoskeletal system [low and upper backache, tension headaches, myofascial pain states, fibromyalgia] or not, there is high contribution of the static load, due to habitual deep muscle tension that adversely affect microcirculation within muscles and the underlying viscera. This is further aggravated by the sedentary life style, lack of exercise, poor posture and distancing from nature [There is high incidence of demineralization of bones due to inadequate exposure to sun].

In contrast to exercises, *asanas* are isometric and can only be studied by subtle electromyographic (EMG) techniques. [There is lower EMG activity in comparison to exercises, which can be further lowered by bringing in awareness and emphasis on relaxation (9)] *Asanas* bring steadiness, health and lightness of the body and optimize tissue functioning. *Asanas* are useful adjuncts in the maintenance phase for reconditioning the body, realignment of skeleton and for correction of postures. It opens up the vital flow of energy through the body, which is subjectively perceived as positive sense of wellbeing. Well chosen culturing and balancing *asanas* can strengthen muscles and correct the posture. This, coupled with relaxation, breaks the cycle and reverses the pain reinforcing forces.

Various *asanas* have compressive / de-compressive effects on the blood flow and lymph flow of underlying tissues through abdominal muscular stretches and contraction in combination with appropriate spinal movements. During acute exacerbations, awareness and progressive relaxation takes away a fair proportion of pain and the person feels more in control, which itself hastens remission.

### Effect of asanas on the neuro-endocrine flow

Asanas stabilize the autonomic nervous system. They influence the endocrine system and nerve plexuses by increasing local blood flow by gravity [*sarvangasana* on thyroid] contraction of surrounding muscle [*Bhujangasana* on lumbar plexus] or by pressure release [*Mayurasana* on celiac plexus].[10] Studies done in regular practitioners show a decrease in cortisol and cholinesterase levels, which reflect quietening of the stress response. There is evidence of endogenous opioid release during sustained stretching of muscles.[11]

### How does Pranayama contribute to management of chronic pain?

*Pranayama* is the controlled expansion of vital energy. In practice, it comprises of yogic breathing techniques; as breath is the one expression of vitality that has both voluntary and involuntary components and multiple associations.

Pain modifies frequency, depth and patterns of respiration. This is due to pain’s emotional component as well as due to the phylogenetically acquired tendency to immobilize the affected area to avoid further injury. If the affected area is localized at the trunk, this may mean considerable restriction of respiratory movements of the chest and/or abdomen.

In chronic pain states, breathing is invariable strained, shallow and mainly thoracic. This is perceived by the physiology as a sustained stressful state and this in turn affects other rhythmic phenomena like neuronal flow and vital cyclic rhythms with high flat cortisol levels similar to arousal response[12,13] Deep yogic breathing with prolonged exhalation relaxes most skeleton muscles. There is some indirect evidence that there is increased tonicity of the parasympathetic nervous system and overall lesser activation of the central nervous system.[14–16]

### Pranayama on cognition states

Just as thoughts and emotions affect the breathing pattern, the converse is also true. Our breathing pattern is reflected internally as stressful or relaxed and can create consequential changes in the physiology. We are familiar with the technique of using slow breathing to come out of stress. Researchers have demonstrated amazing correlations between Electroenchephalography (EEG) patterns and breath patterns. Slow breathing increases the α waves in the EEG and adding feedback of breath sounds further significantly increased it from baseline.[17] Another study elicited a correlation between increase in α waves in the EEG and abdominal breathing,[18] which indicates that states of consciousness could be affected by patterns of breathing. It is impossible to stay angry or anxious while breathing in a slow, deep and reflective manner. Diaphragmatic breathing is probably the single most valuable thing that a patient in chronic pain can learn on the road to recuperation.

## MEDITATION AND PAIN

In general, for most lay people, *yoga* applications are in the above mentioned realms. The last four limbs in the *Ashtanga Yoga* involve the preparatory practices for meditation and meditation. (Beginning with aware introspection [*pratyahara*], there is progressive merging with the nonphysical aspects of our existence through *Dharana, dhyana and samadhi*). The subtler stages require more dedication and hence less prevalent.

*Pratyahara* is the process of graded introspection. *Dharana* [holding together the mind] and *Dhyana* [fixation on a thought] are the progressive stages of meditative practices aiming to eventually merge with the total self awareness [*Samadhi*]. *Pranayama* and meditation appear to work centrally and the effects spread peripherally.

There has been research on different kinds of meditative practices (Transcendental Meditation, guided imagery, progressive relaxation, tai chi, loving kindness etc) and its positive influence on pain states.[19]

### Effects of meditation on physiology and cognition

Neurobiological studies have recognized unique patterns of physiology at different levels of consciousness. Researchers[20] have compared physiological changes seen in supine posture and sleep with that in meditative states. The common findings during meditative states have been much more than just a deeper level of relaxation at physical level (reduced EMG activity in quadriceps, increased finger temperature, pulse volume and circulation, reduction in blood pressure and lactate, constant pCO^2^ and pO^2^ along with significantly reduced O^2^ consumption. The cortisol levels came down while Prolactin and Ach levels increased). Deep meditation has proved to be a hypo-metabolic arousal state.[21] The O^2^ uptake and CO^2^ output are minimal as in deep sleep but the EEG pattern is that of complete alertness. One of the earliest studies in this respect was from AIIMS Delhi.[22] They showed persistent α waves with significant increased amplitude modulation, in the EEG. There is also a tendency towards synchrony of EEG patterns from the various scalp leads.

During self-awareness-based meditation techniques, the person attempts to cultivate the role of an impartial observer and a detached witness of all subjective phenomena including pain. In this way the frame of reference in which pain is experienced, changes. Using mediation to perceive pain may be also considered as a paradoxical technique decreasing the fear of pain.

*Yoga* is thus culturing of the mind and the body.

## RELAXATION RESPONSE

*Asanas* and *Pranayama* initiate a “relaxation response”[23] in the neuro endocrinal system that harmonizes the physiological system. This consists of decreased metabolism,[24] decreased rate of breathing, decreased blood pressure, decreased muscle tension, decreased heart rate and increased slow brain [alpha] waves.[25] As the neural discharge pattern gets corrected, the habitual deep muscle hyper tonicity and thus the static load on postural muscle also slowly come down. The function of viscera improves with the sense of relaxation and sleep gets deeper and sustained. The fatigue level comes down.

Several subtle level notional corrections take place during meditation that changes the context of the disease, pain and the meaning of life. Meditation and *Pranayama* along with relaxing asanas can help individuals deal with the reactive aspects of chronic pain, reducing anxiety and depression effectively.[26]

## APPLICATION IN SPECIFIC CONDITIONS

### Backache

Misalignment of the musculoskeletal components of posture maintenance is found to be the commonest cause for backache. Relaxation *asanas* [*makarasana, shavasana*] followed by stretch [*ardhakati chakrasana, ardhamatsyendrasana*] and strengthening asanas [*Bhujangasana and shalabhasana*] help.[27] Introduction of strengthening asanas too early can aggravate the pain.

*Pranayama* with abdominal breathing initiates relaxation and addition of meditation and awareness maintains it. Gradually, the person becomes aware of his own control over the symptom progression.

### Myofacial pain, Fibromyalgia

Here the primary cause is persistently stressful mindset, which gets reflected on the musculoskeletal system. Fibromyalgia is characterized by chronic muscle tension, systemic fascial adhesion and skeletal malalignment; accompanying is the overactive sympathetic nervous system,[28] inefficient motor activities restricted breathing patterns, fatigueability and low mood.

Yogic practices to regain control include methods of calming, physical self awareness and using minimum muscular effort for optimal results while maintaining full deep breathing that sends signals of relaxation internally. This approach has also been found useful in all other forms of stress -muscle tension – poor blood flow – pain syndromes e.g. tension head ache, RSI

### Migraine[29]

Migraine involves a dysfunctional vascular system and has been associated with personality and stress. Preliminary studies for its control using *yoga* (stretching *asanas* for shoulder, back and neck, whole body relaxation, *jala neti, pranayamas*) show positive results.

Yogic breathing is a unique method, which helps balance the autonomic nervous system and influence psychological and stress-related disorders[30] Mechanisms contributing to a state of calm alertness include increased parasympathetic drive, calming of stress response systems, neuroendocrine release of hormones, and thalamic generators.

### Knee pain

Osteoarthritis (OA) is the most common form of arthritis and a leading cause of disability. As there are no disease-modifying agents available for OA, the standard care of patients with osteoarthritis of the knees is aimed at reducing pain and improving the functioning of the knees by focusing on the surrounding tissues through exercise or external support. Regular *pawanamukthasana* [anti-rheumatic series] or BKS Iyengar (*Padmavibhushan* BKS Iyengar – is a proponent of a method of *yoga* named after him. His school is based in Pune, Maharashtra.) Yogic techniques[31] have been successfully used to contain the symptoms and disability. These are easily learnt techniques for regular mobilization of each joint of the body synchronized with breathing and awareness.

### Neuropathic pain

Neuropathic pain is caused by nerve damage proximal to the sensory nerve endings in the skin. It has no protective or predictive value and inflicts significant sensory and emotional burdens on. Most neuropathic pain patients experience some improvement when physicians adopt a holistic approach to treatment. Components used from *yoga* are, *yoga nidra* with visualization, breath and body awareness, gentle equivalent stretch *asanas* for muscle groups and meditation.

There have been studies on successful use of upper body postures: improving flexibility; correcting alignment of hands, wrists, arms, and shoulders; stretching; and increasing awareness of optimal joint position during use to alleviate symptoms of specific areas like carpal tunnel syndrome.[32]

### Cancer pain

Pain in cancer is a body-mind phenomenon and obviously requires therapy directed to both. The commonest techniques used are gentle regular mobilization through *pawanamuktasana*, which can be performed even by a bedridden patient, specific calming *pranayamas* and *yoga nidra* with visualization to sublimate the suppressed emotional components. If the patient is committed, meditation can be included. In malignant pain states, relaxation postures and *yoga nidra* (guided progressive deep relaxation technique which works in alignment with the sensorimotor cortex representation of the body) have been found to reduce analgesic requirements and improve sleep and reduce fatigue.

Besides improvement in the pain experience, there is significant improvement of other distresses including fatigue[33] insomnia and breathlessness. The “dissolution of body” experience and the oneness, that the *pranayama* and meditative methods provide allows an experience of healing and wholeness.[34] Increased self-understanding and self-acceptance, changed context of pain, increased control, life style improvements, group and social support involved in undertaking yogic activities all together contribute to improved quality of life.[35]

## DISCUSSION

Life is but a string of experiences consisting of perceptions, interpretation and behavior patterns. We have learnt the modalities of sensory perception in detail, that of stimulus, receptor, action potentials, nerve pathways, synapses, transmitters, central processing followed by perception and interpretation. But, is this complete? Has it provided us with the wisdom to sufficiently handle abnormalities in the same?

For example, if this was all that perceptual experience was about, then what happens under general anesthesia or in comatose states? Each and every component of perception mentioned above, is intact and available in the body, yet we don’t feel a knife cutting through.

Most of the information basis of our medical training has been structure based, and acquired through in vitro experiments, animal studies or from dead bodies, where the fine web of intelligence/consciousness is altered or absent. The teachings, thus, do not elaborate much about the life principle behind each perceiving sense organ. This is the principle that the traditional sciences talk about, which enlivens the human being to perceive and experience.

Let us humbly acknowledge that there may be huge gaps in modern medicine regarding the knowledge and understanding of human experiences, and the pathological processes affecting it.

*Yoga* [oneness, unity] is the foremost science of self realization and right living. It is described as that which sublimates and dissipates the turbulences within. Through various methodologies, it evokes a poise of the soul which enables one to look at life in all its aspects evenly. The principles strive to bring balance and harmony in all aspects of existence.

*Yoga* uses the model of five-dimensional perceptions.[36] The human being is considered to experience every interaction through the physical, vital, emotional, intellectual and spiritual perceptions simultaneously, with the physical sheath being the grossest and others progressively subtler; and the intellectual and spiritual perceptions being the subtlest. It also states that the ripples in the emotional sheath affect the grosser dimensions of vital energy flow and physical body.

## CONCLUSION

Research is accumulating evidences, acknowledging human existence beyond physical terms as shown by neurobiological studies at different levels of consciousness,[21] development of psycho neuro immunology[37] as the most happening field in science and studies on “relaxation response”[23] The definitive influence of communication in symptom management is being acknowledged.[38]

The culturing of the body and mind that happens through regular *yoga* inputs brings about an equanimity of perception, which makes conventional inputs including analgesics more effective in controlling the distress of chronic pain.

Quality of life has been described through the “gap theory” as the discrepancy between an individual’s expectations and perceptions of a given situation- the smaller the gap, greater the quality of life.[39] *Yoga* is one input which effectively brings down this gap by influencing both the components of the ratio. Let us learn and utilize the components of this ageless science to evoke the inner resources of our patients suffering from chronic pain, and calm the multi-dimensional eddy currents of distress to improve the quality of life and achieve “freedom from pain”.
